# Meeting of the American Pharmaceutical Association

**Published:** 1875-09

**Authors:** 


### Meeting of the American Pharmaceutical Association.

The recent meeting or this Association, which was held in Boston, was largely attended and full of interest. The address by the President, C. Lewis Diehl, of Louisville, Ky., was full of interest. Among other things he referred to the new remedies recently introduced, jaborandi and salicylic acid. Among the interesting reports was one by Dr. A. W. Miller, of Philadelphia, on adulterations and sophistications of Drugs. He said that essential oils were largely adulteradated, often in a clumsy manner. A large number of instances were cited which had been brought to the notice of the committee in which important drugs had been largely adulterated. This is a matter of great interest to physicians as well as to pharmacists, and something should be done to punish the offenders. The report on the Progress of Pharmacy was a very elaborate essay from the President, Mr. C. Lewis Diehl, more extensive it is said, than the one made by same gentleman last year which occupied over three hundred pages in the volume of proceedings. A large number of interesting essays and reports were made. The exhibition of rare drugs, chemicals and improved laboratory apparatus was very large and valuable.

The members were handsomely entertained by their brethren in Boston, who treated them to banquets, hops, concerts, &c., in profusion. The Association meets on the second Tuesday in September, 1876, in Philadelphia.

---

Announcement.—Under the head of Medical Notes Dr. George E. Blackham, of Dunkirk, has kindly consented to furnish the Journal with occasional notes on Microscopy. Dr. Lucien Howe, of this city, will also contribute notes on the progress in Ophthalmology. It is our intention to make this department of the Journal a record of the progress in the various branches of medical science, and to this end shall spare no pains to make it interesting and instructive.——Opening of the Lecture Course.—The course of lectures in most of the medical colleges have already commenced or will do so in a few weeks. The preliminary term at the Buffalo Medical College will open Wednesday, Oct. 6th, the regular term term on the 3d of November. The Medical and Surgical Clinics and Lectures on special topics will be given during the preliminary term.——Chicago Medical Journal and Examiner.—The *Chicago Medical Journal* and the *Medical Examiner* have been united under the above head. The new Journal is published by the Chicago Medical Press Association and is edited by Wm. H. Byford, M. D., assisted by Drs. Jas. H. Etheridge, Norman Bridge, J. N. Hyde and F. C. Hotz.——New Chair.—The Starling Medical College, Columbus, Ohio, has a Professor of Ovariotomy.——Code of Ethics.—The Code of Ethics of the American Medical Association has been unanimously adopted by the Medical Society of Munich, and translated into the German language.——Syphilitic Ghost.—Prof. Ziessl, of Vienna, is reported to have said in a clinic "that if a man contracts syphilis, he will die syphilitic, and at the judgment day his ghost will have syphilis."

